# Bayesian adaptive model estimation to solve the speed accuracy tradeoff problem in psychophysical experiments

**DOI:** 10.1038/s41598-021-97772-9

**Published:** 2021-09-14

**Authors:** Jongsoo Baek, Hae-Jeong Park

**Affiliations:** 1 Center for Systems and Translational Brain Sciences, Institute of Human Complexity and Systems Science, Yonsei University, Seoul, Republic of Korea; 2 Department of Nuclear Medicine, Department of Psychiatry, Yonsei University College of Medicine, Seoul, Republic of Korea; 3 Graduate School of Medical Science, Brain Korea 21 Project, Yonsei University College of Medicine, 50-1 Yonsei-ro, Sinchon-dong, Seodaemun-gu, Seoul, 03722 Republic of Korea; 4 Department of Cognitive Science, Yonsei University, Seoul, Republic of Korea

**Keywords:** Human behaviour, Computational models

## Abstract

Most psychological experiments measure human cognitive function through the response time and accuracy of the response to a set of stimuli. Since response time and accuracy complement each other, it is often difficult to interpret cognitive performance based on only one dependent measurement and raises a speed-accuracy tradeoff (SAT) problem. In overcoming this problem, SAT experimental paradigms and models that integrate response time and accuracy have been proposed to understand information processing in human cognitive function. However, due to a lengthy SAT experiment for reliable model estimation, SAT experiments' practical limitations have been pointed out. Thus, these limitations call for an efficient technique to shorten the number of trials required to estimate the SAT function reliably. Instead of using a block's stimulus-onset asynchrony (SOA) accuracy with long block-based task trials, we introduced a Bayesian SAT function estimation using trial-by-trial response time and correctness, which makes SAT tasks flexible and easily extendable to multiple trials. We then proposed a Bayesian adaptive method to select optimal SOA by maximizing information gain to estimate model parameters. Simulation results showed that the proposed Bayesian adaptive estimation was highly efficient and robust for accuracy and precision of estimating SAT function by enabling "multiple-step ahead search."

## Introduction

In many psychophysical experiments (e.g., Stroop, Flanker), the primary measurements for behavior performance are response speed and accuracy. Despite specific information on each measure, researchers often focus on one measurement type while ignoring the other. However, response speed usually depends on accuracy, for example, the shorter response time (RT) with lower accuracy or vice versa. Thus, ignoring one aspect makes it difficult to interpret the other result correctly. This is called the speed-accuracy tradeoff (SAT). Inter-dependency between speed and accuracy should be considered to understand human cognitive processing.

Further, a simple RT or accuracy measurement does not provide insight into the accumulation of information over time. When there is no temporal constraint for a response, participants tend to respond to the stimulus when confident about their responses. That is, most responses are made with sufficient RT and high accuracy. Thus, experimental data do not include accuracy for quick responses (i.e., cognitive performance when the internal processes are occurring).

To study additional details about human information accumulation, researchers have developed the SAT experiment, a class of experimental manipulations by spreading or limiting RT over a wide range of time, and measurement accuracy as a function of RT. In a typical SAT experiment, participants are asked to change the strategy for decision-making and responding, emphasizing either fast or accurate response—with various cues: verbal instruction, payoffs, response deadline, or/and response-signals. For example, with the response-signal manipulation^1^, participants can respond only during a response-signal with a certain duration being given. Each response-signal is presented for every trial or block after diverse stimulus-onset asynchrony (SOA)—the elapsed time between stimulus onset and response-signal. The stimulus was selected with the method of constant stimuli (MCS), where a fixed set of SOA is predetermined by the experimenter and repeatedly presented in random order of SOA blocks.

Participants should respond quickly in short SOA conditions but have enough time to prepare responses in long SOA conditions. In this manipulation, mean RT and accuracy can be calculated for each SOA condition, and mean RT is plotted against accuracy. The SAT function, the accuracy as a function of mean RT, is known to follow a conditional accuracy function form:

1 $$ {\text{p}} = \lambda \left( {1 - {\text{exp}}^{{ - \gamma \left( {{\text{T}} - \delta } \right)}} } \right) + {\upmu }\;{\text{for}}\; \, T > \, \delta ,\;{\text{otherwise}}\;p \, = \, 0 $$

where p is accuracy, λ the asymptotic performance, γ the rate parameter for the change of accuracy as a function of T, δ the x-axis intercept and ${\upmu }$ an offset in the y-axis where accuracy begins to rise above chance performance (i.e., d′ = 0), and T is response time^2^. For example, in a two-alternative forced-choice task in which the chance level is 0.5, the SAT function can be expressed by:

2 $$ {\text{p}} = \lambda \left( {1 - {\text{exp}}^{{ - \gamma \left( {{\text{T}} - \delta } \right)}} } \right) + 0.5 $$

where p is probability correct. Mean RT and accuracy are fitted to the model with maximum likelihood method or least square method to estimate SAT function parameter.

**Figure 1:**
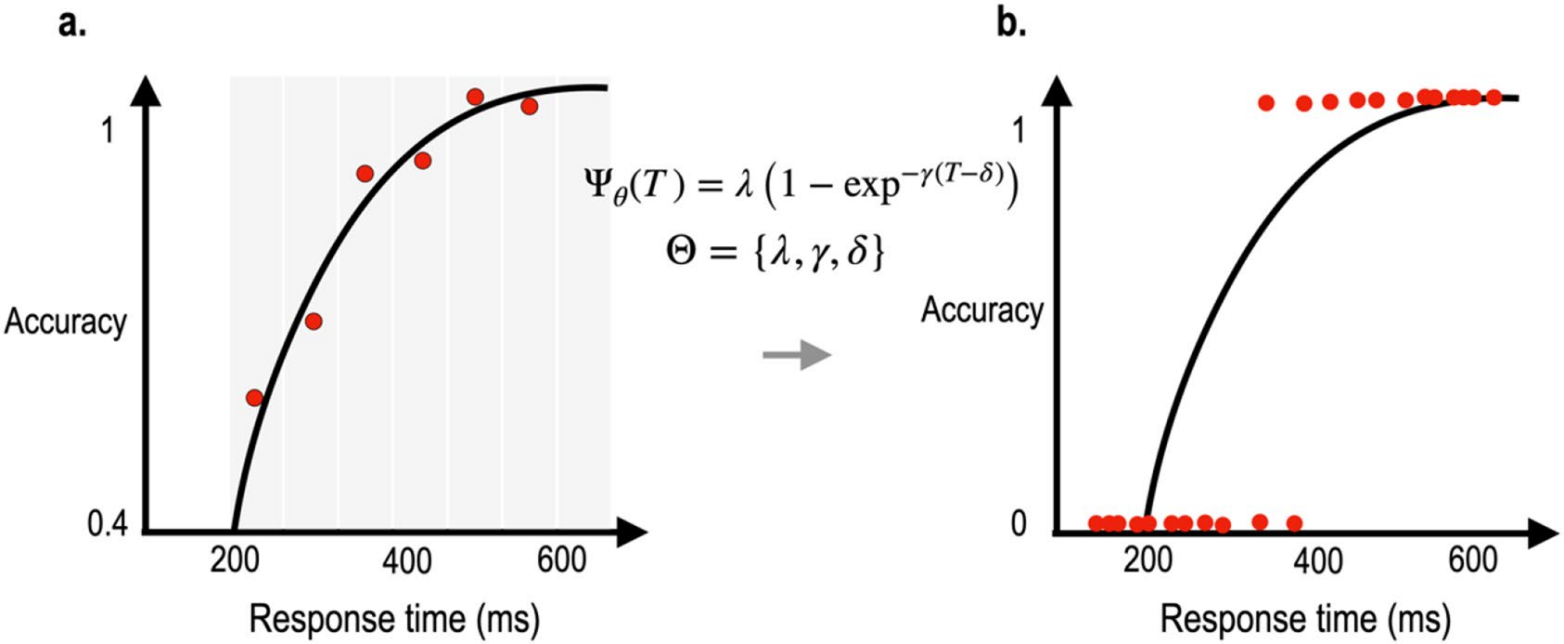
The binary decision problem for SAT. (**a**) Conventional SAT function estimation using a limited number of SOA (response time) bins and high-precision response accuracies for those bins. (**b**) Current SAT function estimation using responses' binarized correctness at various response times or SOAs.

Although SAT function provides detailed information about cognitive processes by measuring performances at multiple processing time points, trial numbers and experimental time for measuring SAT function is often demanding. This is because data from diverse SOA levels are required to fit the SAT curve and because the accuracy at each SOA level can be reliably evaluated with enough trials for the SOA (Fig. 1a). Indeed, SAT experiments are mainly based on long blocked trials to achieve sufficient precision for different SOA levels.

Since the SAT procedure requires sufficient data collection for multiple data points (i.e., SOA level), experimental time could be several times longer in SAT experiment than in the non-SAT experiment. For example, a non-SAT version of the Flanker experiment collects 100 responses without restricting response times; an experiment with SAT manipulation could require 800–1000 trials, 100 trials for each of the eight SOA levels. If the research design includes other variables (e.g., congruency), experimental time gets even longer. The burdensome data collection blocks the popularity of SAT experiments in psychological laboratories. Furthermore, it is not easy to run such experiments for special populations who have problems maintaining attention for a long time, such as patients, the elderly, or children. Thus, optimizing the SAT procedure is required for a quick and efficient estimation of the SAT function.

To make it efficient in data acquisition, we introduced an adaptive approach to estimate SAT function, which has often been used in the cognitive model parameter estimation^3–8^. The adaptive design recursively processes the model parameters and optimal stimulus selection based on the previous and real-time response data. The optimal stimulus is determined to maximize information gain to estimate the unknown model parameter reliably.

For the adaptive approach, a long block-based conventional SAT task, which is inevitable for evaluating accuracy for the SOA of the block, may not be appropriate. Thus, estimation of the SAT function in an adaptive way calls for manipulation of SOA for a single trial or shorter blocked stimulation. In the current paper, we propose a single trial-based estimation of SAT with diverse SOA (Fig. 1b). In the conventional block-based SAT experiment, the number of SOA levels is often limited to 5–10 conditions since the number of stimuli should be high enough to calculate accuracy with sufficient precision for each SOA level. Thus, the blocked approach results in a lower resolution on the temporal scale (several bins of SOA), while higher resolution in the accuracy.

Therefore, we developed a method to estimate SAT function based on a single trial or a short block (batch) trial data for an SOA by utilizing data for diverse SOA levels. In this approach, a high temporal resolution compensates for the lower resolution of accuracy in estimating the SAT function. The advantage of a single-trial or shorter block approach we prefer is its adequacy in terms of the adaptive design. Based on the single trial-based method, we propose an adaptive stimulation technique to estimate the SAT function.

**Figure 2:**
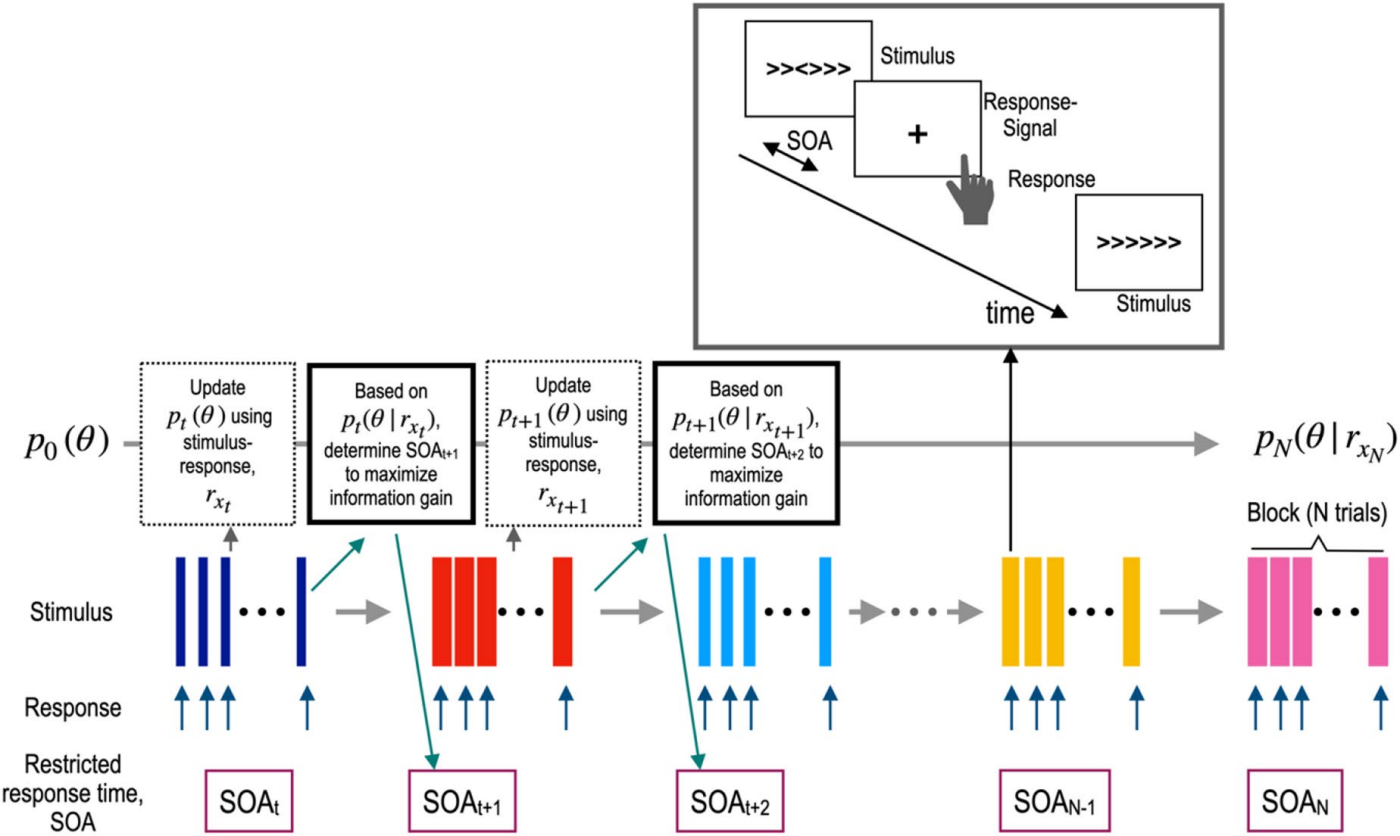
The overall procedure of the Bayesian adaptive estimation for SAT function. From a prior ${p}_{0}\left(\theta \right)$, model parameter $\theta$ is estimated in a Bayesian update rule. After the t-th trial, the prior distribution ${p}_{t}\left(\theta \right)$ at the t-th trial is updated to the posterior distribution ${p}_{t}(\theta |{r}_{x})$ with the observer's binarized response ${r}_{\mathrm{x}}$ (correct or incorrect) for a stimulus with an RT *x* by Bayes rule. See Method section for details.

The current paper is composed of three main parts. First, we describe a mathematical formulation for the single trial-based SAT function estimation. Second, we present a Bayesian formulation for the adaptive design technique to estimate SAT function. Third, we present simulations for the trial-based estimation and adaptive design technique and evaluate its performance compared to the convention SAT parameter estimation technique. To be more specific, we compared four SAT model estimation techniques with (1) conventional maximum likelihood method (procedure 1), where SAT model was fitted to simulated data with mean RT and accuracy for each block with a different SOA; (2) maximum likelihood estimation (procedure 2), where trial-by-trial RT and correctness was used for model fit, without averaging RT and correctness by SOA or RT; (3) Bayesian parameter estimation using trial-by-trial data (procedure 3) as a Bayesian version of procedure 2; and (4) Bayesian adaptive parameter estimation using trial-by-trial data (procedure 4) as an adaptive version of procedure 3. The procedures 2, 3, and 4 we introduced in this study are separately evaluated to show the performance increase according to additive features in the model estimation. Figure 2 explains the Bayesian adaptive estimation scheme for SAT function used in the current simulation.

We expect the current approach to providing a new practical scheme to explore human performance concerning speed and accuracy.

## Results

Results showed that the procedure for fitting trial-by-trial data using Bayesian estimation with optimal stimulus selection was the most efficient and robust for accuracy and precision of estimating SAT function and parameters.

### The Bayesian adaptive procedure with optimal stimulus selection

**Figure 3:**
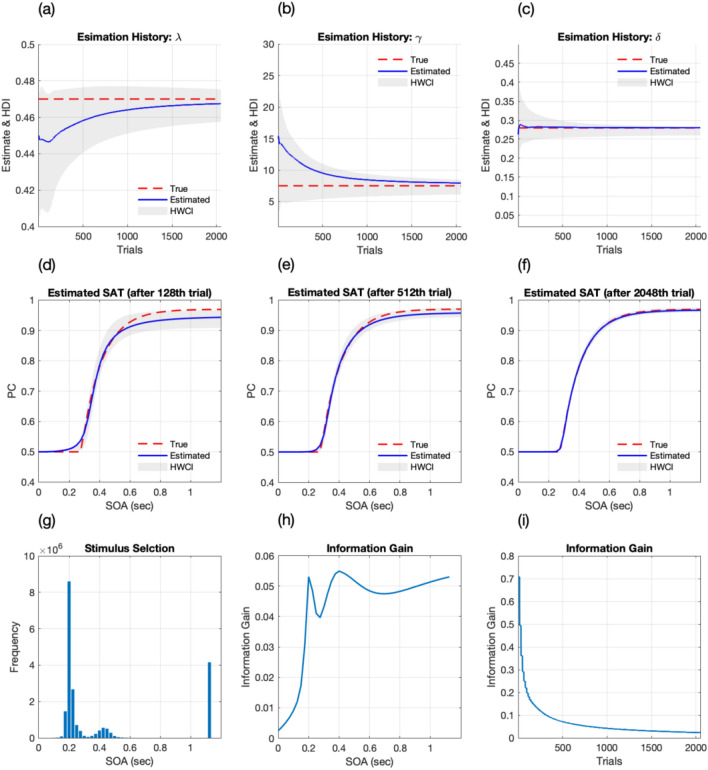
Results of simulations with the Bayesian adaptive estimation (Procedure 4). (**a**–**c**) Accuracy and precision of λ, γ, δ estimation. The mean of estimated parameters (blue curve) approached the true parameter (red line). HWCI of estimates also quickly decreased as trials were iterated. (**d–f**) Accuracy and precision of the estimated SAT function were obtained with 128, 512, and 2048 trials. With less than 128 trials, the procedure recovered the general shape of the SAT function. Further, it increased both accuracy and precision (**g**) stimulus selection occurred intensively in three SOA ranges: short (0.2–0.3 s), mid (0.35–0.45 s), and long (1.2 s) (**h**) information gain over SOA was maximum at the three SOA ranges where the stimulus was frequently selected. (**i**) Information gain was much higher in earlier trials than later trials.

Figure 3a–c show the history of parameter estimates (λ, γ, δ respectively) for the virtual observer 1 with Procedure 4. The mean estimates of parameters (blue) converged to the true parameter (red), and the precision of the estimated parameters increased with the trial number. Shaded areas represent 68.2% of the half-width of the credible interval (HWCI) averaged over simulations. For λ, the average bias became less than 0.023 after 128 trials, and 0.021 after 256 trials, and further decreased to 0.017 after 512 trials, 0.012 after 1024 trials, and 0.007 after 2048 trials. The average bias for γ was 5.322 after 128 trials, 4.071 after 256 trials, and further decreased to 2.626 after 512 trials, 1.586 after 1024 trials, and 0.983 after 2048 trials. For δ, the average bias was 0.033 after 128 trials, and 0.025 after 256 trials, and further decreased to 0.018 after 512 trials, 0.013 after 1024 trials, and 0.008 after 2048 trials. The average 68.2% HWCI of λ started at 0.035 in the first trial, and decreased to 0.032 after 128 trials, 0.018 after 512 trials, and 0.009 after 2048 trials for λ; started at 9.752 in the first trial, and decreased to 5.481 after 128 trials, 2.742 after 512 trials, and 1.164 after 2048 trials for γ; started at 0.135 in the first trial, and decreased to 0.042 after 128 trials, 0.022 after 512 trials and 0.011 after 2048 trials for δ.

Figure 3d–f shows the accuracy and precision of the estimated SAT functions obtained with 128, 512, and 2048 trials. The true SAT functions are plotted as red curves, and the estimated functions are shown as blue curves. Shaded areas represent the 68.2% HWCI of the estimated functions over simulations. With increasing trial numbers, the procedure improved accuracy (decreasing discrepancy between blue and red curves) and precision (reducing shaded area) of the estimated SAT functions. It took less than 100 trials to recover the SAT function's general shape (average absolute bias = 0.020) and decreased to below 0.019 after 128 trials, 0.011 after 512 trials, and 0.005 after 2048 trials. The average 68.2% HWCI became less than 0.021 after 128 trials, and decreased to below 0.012 after 512 trials and 0.006 after 2048 trials. The results indicate that Procedure 3 can rapidly estimate the true SAT function with only a small number of trials compared to the conventional SAT procedure.

Stimulus sampling pattern of the procedure for observer 1 is presented in Fig. 3g. The procedure intensively tests three ranges of SOA: short (0.2–0.3 s), mid (0.35–0.45 s) and long (1.2 s) SOAs to characterize δ, γ and λ. The procedure does not frequently test SOA greater than 0.5 s (except the longest SOA, 1.2 s) throughout the whole experiment. Figure 3h shows the information gain as a function of SOA accumulated over entire trials. The information gain was higher in the three ranges that the stimulus was selected frequently. Figure 3i shows the trial-by-trial information gain. It is evident that the procedure gains much more information in earlier trials than later trials.

**Figure 4:**
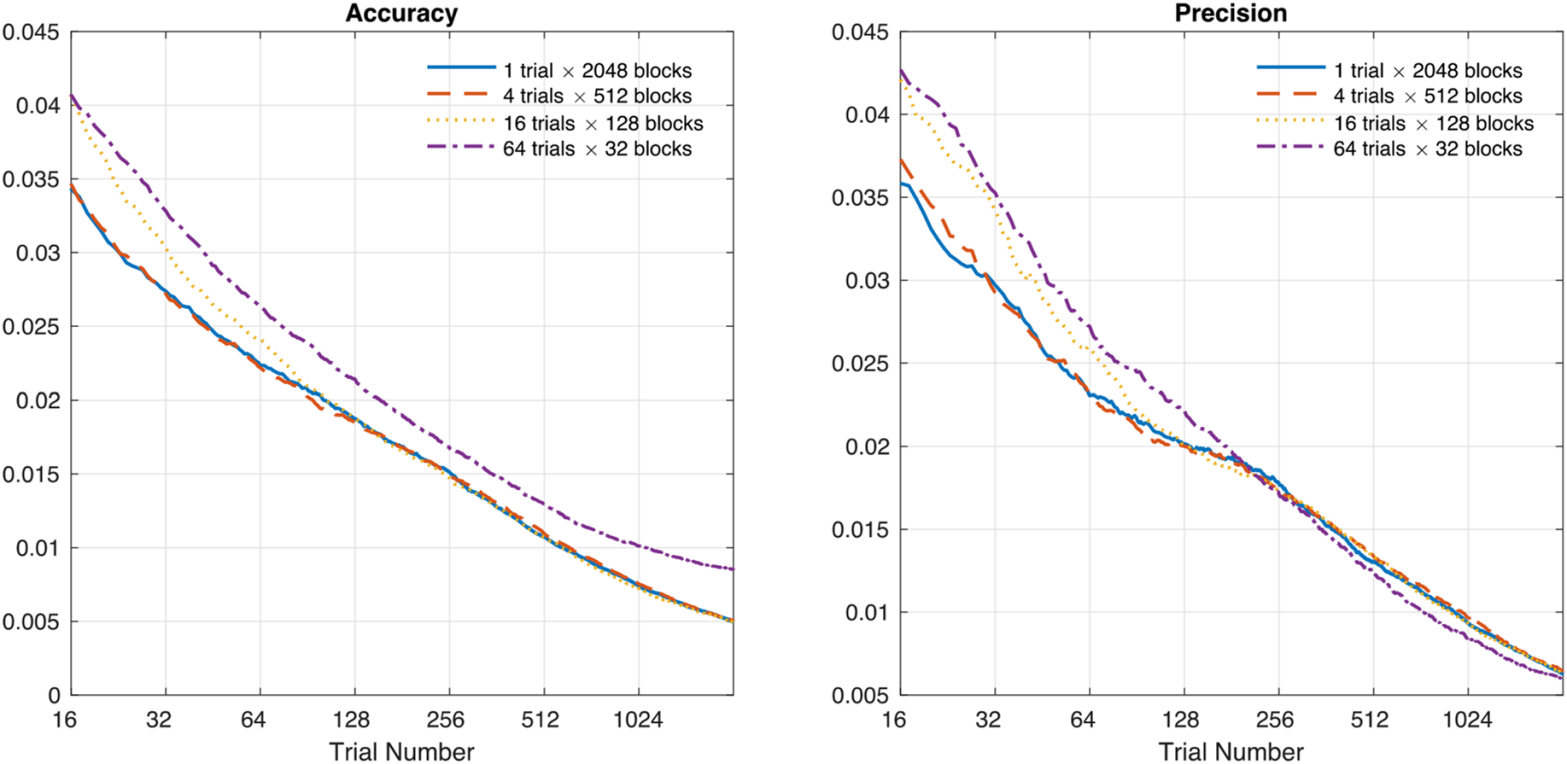
Accuracy and precision of SAT function estimated with Procedure 4 with different block lengths (1, 4, 16, and 64 trials in each block). The procedure with smaller block lengths (1 and 4) showed better accuracy and precision than with larger block lengths when a total number of trials was small (< 128 trials). The accuracy and precision with block length = 16 trials converged to those with smaller block lengths after 128 trials.

The performance of the proposed procedure would vary with the number of trials in each block, *block lengths*. To explore the effects of block length (i.e., the number of trials in each block) on the performance of Procedure 4, we simulated responses of observer 1with four different block length settings: 1, 4, 16, and 64 trials in each block. Simulations were iterated 1,024 times for each setting. As shown in Fig. 4, the procedures with smaller block lengths (i.e., block size = 1 or 4 trials) generally showed better accuracy than others. The accuracy of the procedure with block length = 16 trials converged to that with smaller block lengths after a small number of total trials (e.g., after 128 trials, bias was 0.019, 0.019, and 0.019 for the procedure with block length = 1, 4, and 16 respectively). However, the accuracy with block length = 64 trials (bias = 0.021 after 128 trials) did not converge to those with smaller block sizes even after a large number of trials.

The precision of the procedure also varied with the settings of the block length. The precision of the procedure with block length = 16 was worse than those with smaller block lengths at the beginning but became almost equivalent to after a small number of total trials (SD = 0.020, 0.020, and 0.020 for the procedure with block length = 1, 4, and 16 respectively after 128 trials). The precision with block length = 64 trials (SD = 0.022 after 128 trials) was greater than those with smaller block lengths when a total number of trials were relatively small, but converged to others after 256 trials (SD = 0.018, 0.017, 0.017, and 0.017 for the procedure with block length = 1, 4, 16, and 64 respectively).

### Accuracy

**Figure 5:**
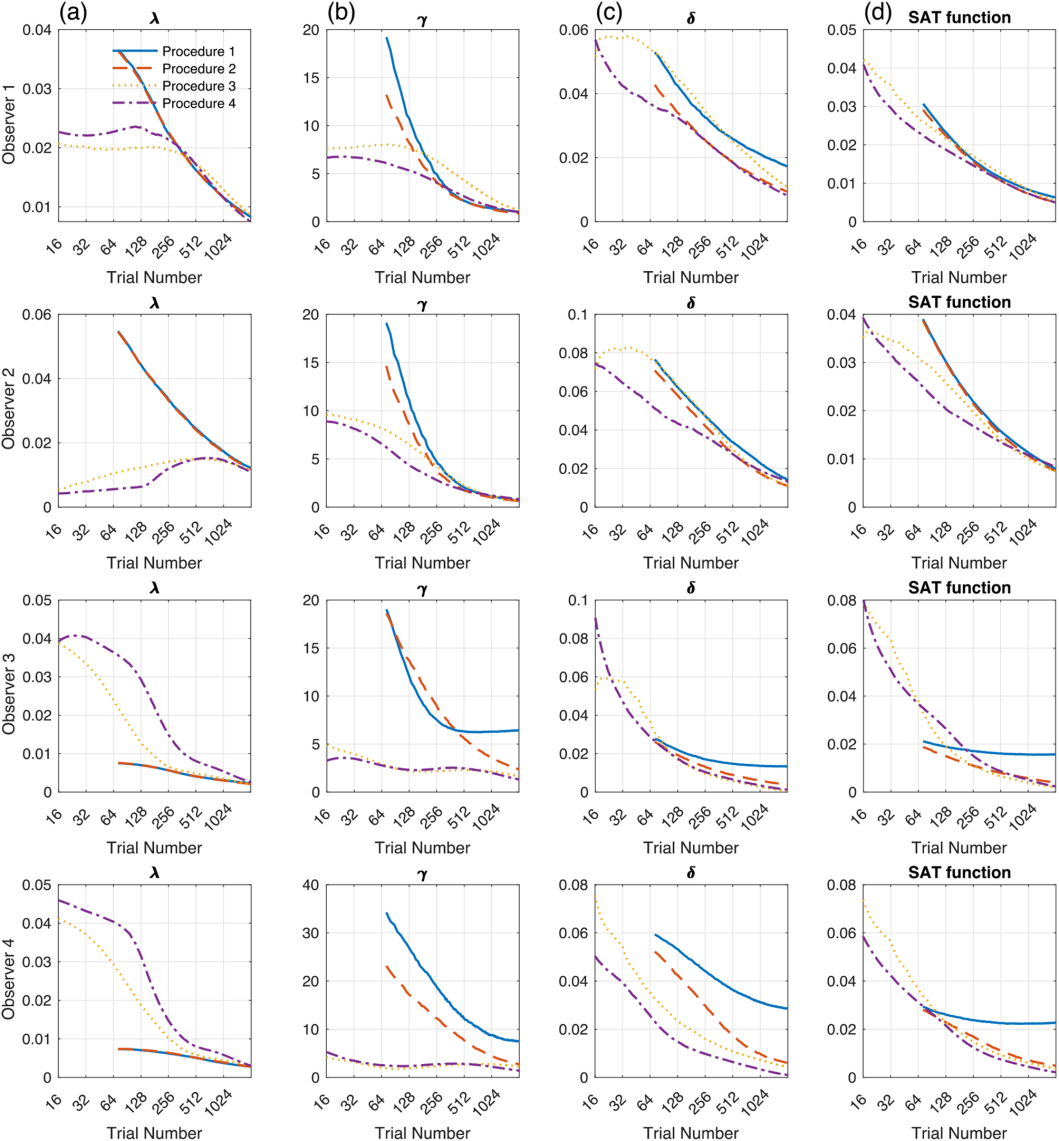
The accuracy of estimated SAT parameters and function with four estimation procedures for four simulated participants. The bias (mean absolute difference between true and estimated parameters) of (**a**) λ, (**b**) γ, and (**c**) δ was plotted as a function of trial numbers. In each panel, different procedures were presented with different colors. Procedure 4 showed better or equivalent accuracy (more negligible bias) than the other procedures, except λ for participants 3 and 4. (**d**) Mean absolute difference between true and estimated SAT function was also smaller with Procedure 4 than with the other procedures. (*Note.* Procedure 1: Maximum likelihood estimation with mean RT and accuracy; Procedure 2: Maximum likelihood estimation with trial-by-trial RT and accuracy; Procedure 3: Bayesian estimation without optimal stimulus selection; Procedure 4: Bayesian adaptive estimation with optimal stimulus selection).

Figure 5 shows the accuracy of estimated SAT parameters and function for the four simulated observers over 2048 trials. The bias of estimated parameters—the mean absolute difference between true and estimated parameters—was plotted against the number of simulated trials with four estimation procedures in the first three columns (λ, γ, δ for column a–c, respectively).

Generally, the bias of parameter estimates decreased as trial repeated in all observers. For λ, Procedure 1 and 2 showed almost equal bias, but higher than Procedure 3 and 4 in observers 1 and 2 and lower in observers 3 and 4. For γ, there was a difference in bias between Procedure 1 and Procedure 2 for observers 3 and 4 who have relatively high γ values. For observer 3, the bias of estimated γ was saturated higher in Procedure 1 than Procedure 2 and did not decrease after 500 trials. For observer 4, Procedure 1 showed higher bias than Procedure 2 over whole trial numbers. Procedure 3 and 4 were superior to Procedure 1 and 2 in all observers, especially when a small number of trials was tested. In all observers, Procedure 4 showed similar or slightly better accuracy than Procedure 3. A similar pattern was observed in the bias of estimated δ: smaller bias with Procedure 4 than with Procedure 1 and 2. Accuracy of Procedure 3 was close to Procedure 1 and 2 in observer 1–3, but to Procedure 4 in observer 4.

The rightmost column in Fig. 5 shows the accuracy of the estimated SAT function. The bias of estimated functions rapidly decreased with trial numbers tested. For observers 1 and 2, accuracy was comparable between Procedure 1 and 2, but better with Procedure 3 and 4 than Procedure 1 and 2. For observers 3 and 4, there was a significant difference in bias between Procedure 1 and Procedure 2–4. Therefore, in terms of accuracy of estimation, we conclude (1) that the fitting trial-by-trial procedure (Procedure 2) was superior to the conventional procedure of fitting averaged data (Procedure 1), since it was more robust for estimating SAT function and parameters for observers with high γ value and (2) that the Bayesian adaptive procedure with or without optimal stimulus selection (Procedure 3 and 4) is comparable or better than the fitting trial-by-trial procedure (Procedure 2).

### Precision

**Figure 6:**
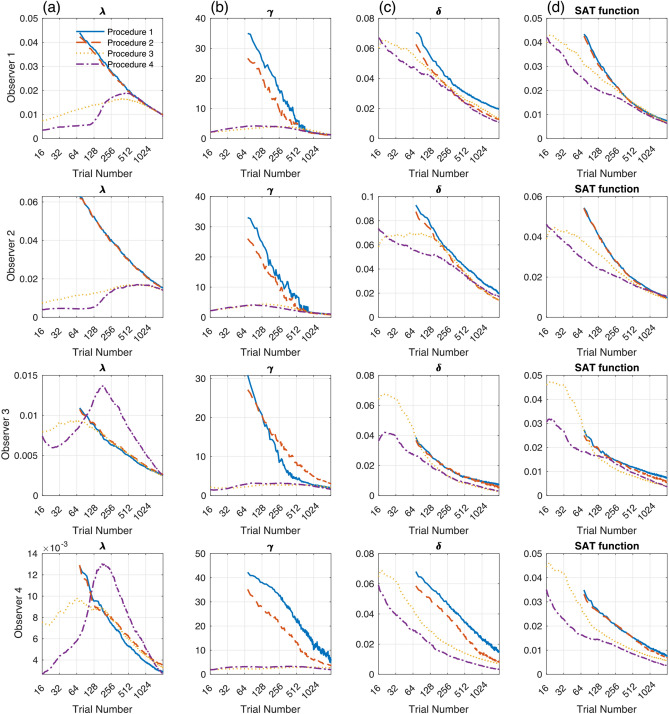
The precision of estimated SAT parameters and function with four estimation procedures for four simulated participants. The standard deviation of (**a**) λ, (**b**) γ, and (**c**) δ estimates was plotted as a function of trial numbers. In each panel, different procedures were presented with different colors. Procedure 4 showed better precision (smaller standard deviation) than the other procedures, especially for a small number of trials. (**d**) The standard deviation of the estimated SAT functions was also smaller with Procedure 4 than with the other procedures. (*Note.* Procedure 1: Maximum likelihood estimation with mean RT and accuracy; Procedure 2: Maximum likelihood estimation with trial-by-trial RT and accuracy; Procedure 3: Bayesian estimation without optimal stimulus selection; Procedure 4: Bayesian adaptive estimation with optimal stimulus selection).

Figure 6 shows the precision of estimated SAT parameters and function with four estimation procedures. With Procedure 1 and 2, the standard deviation of estimated parameters over 10,000 simulations decreased as trial number increased. With Procedure 3 and 4, the standard deviation for λ and γ increased initially but dropped after a small number of trials. The smaller standard deviation at the beginning of the Bayesian estimation procedures is caused by the same prior distribution in repeated measures: the procedures always start with the uniform prior distribution for all simulations. For all parameters, Procedures 1 and 2 showed comparable precision but worse than Procedures 3 and 4. It should be noted that precision for λ was slightly smaller with Procedure 1–3 than with Procedure 4 for observers 3 and 4, but the difference was minimal (less than 0.006). The superiority of precision with Procedure 4 was more significant in smaller trial numbers.

Procedure 4 also showed the best precision of the estimated functions. Procedure 1 and 2 showed comparable precision but worse than Procedure 3 and 4. With Procedure 4, the average standard deviation of the function became less than 0.02 after 95 trials on average (i.e., about 6 blocks of 16 trials). To reach a 0.020 probability correct precision, Procedure 1 requires 304, 512, 136, and 272 trials for observers 1–4, respectively. However, Procedure 4 only needs 132, 273, 53, and 40 for observers 1–4, respectively. It was about 2–6 times more efficient than Procedure 1 (The ratio of trials required for 0.02 precision with Procedure 4 vs. Procedure 1 is 0.434, 0.533, 0.390, and 0.147). Results indicated that the Bayesian adaptive procedure for trial-by-trial data with optimal stimulus selection (Procedure 4) could rapidly estimate the true SAT function for a wide range of potential populations with reasonable accuracy and precision within only 15–50% of the experimental time of the conventional procedure with MCS and MLE for averaged data (Procedure 1).

## Discussion

In the current study, we suggest two methods for adaptively estimating the SAT function with smaller samples (i.e., number of trials). We firstly introduce a Bayesian inference for estimating SAT function using trial-by-trial RT and binarized correctness. The trial-by-trial RT and binarized correctness can be extended to deal with trial RTs and correctness within a short or long block. We then introduce an optimal adaptive scheme to select the most informative stimulus level for the next block. Starting with a prior distribution of the parameters, the Bayesian adaptive procedure tests the observer at the most informative SOA by evaluating the stimulus space to find the SOA that maximizes the expected information gain or minimizes the entropy of the posterior distribution of the SAT function parameters. It then updates the probability distribution of the parameters based on the observer's response by Bayesian inference. The procedure is iterated until the total number of trials reaches a set value. Compared to the conventional SAT procedure with MCS, the Bayesian adaptive SAT procedure uses a much finer stimulus sampling resolution. It estimates the SAT function's whole shape with much less testing since it concurrently measures the observer's performance across all different SOA conditions and utilizes all available information acquired during the experiment and prior knowledge about the functional form of the SAT function. Results from simulations showed that the procedure requires only 50–150 trials of data collection to measure the SAT function with reasonable accuracy and precision: with only 100 trials, the procedure achieved 0.024 average absolute bias and 0.020 precision (in probability correct). That is, with this procedure, reasonably precise estimates can be obtained in 5–10 min, which is significantly less than the typical one-hour test time for the conventional MCS. We believe that the proposed procedures can work as a valuable tool in many clinical settings and cognitive laboratories.

Several Bayesian adaptive procedures have been developed for many psychophysical experiments^6,7,9–11^. QUEST, the first Bayesian adaptive procedure in psychophysics^11^, was developed for threshold estimation of the psychometric function, which follows Weibull distribution. The Φ procedure^6^, which estimates the threshold and slope of psychometric function concurrently, further optimized the experimental procedure by adopting the stimulus selection with minimum entropy^6,7,9,10^ . Researchers applied the algorithm of the Φ procedure to the estimation of other psychological functions. For example, qCSF estimates contrast sensitivity functions^10,12–14^, qYN assesses sensitivity and response bias in Yes–No task^15^, and qPR examines iconic memory decay function^3^. These applications can estimate multiple parameters and select optimal stimulus in multi-dimensional stimulus space (e.g., qCSF estimates three parameters with selecting contrast and spatial frequency of the next stimulus). Although the specific algorithms have become more complex and sophisticated, the algorithms' core was the same for all these procedures.

Our development of the SAT function adopted the algorithm from previous Bayesian adaptive procedures. However, there are some significant aspects of this procedure. First, our procedure is an adaptive procedure that can handle RT data. While all the previous Bayesian adaptive procedures focused on the fitting accuracy for pre-determined stimulus levels ignoring RT, our procedure fit accuracy for participant's RT. Therefore, the estimated SAT function enables us to understand more about the underlying cognitive process. Second, in our procedure, we implemented a block-based optimal stimulus selection. Most Bayesian adaptive procedures for psychophysics change stimulus level trial-by-trial with "one-step-ahead search"—finding the "current best" stimulus for the subsequent trial. Conversely, by its nature, the SAT experimental procedure should change the stimulus by block. Due to this unique SAT procedure feature, optimal stimulus selection should be made by blocks with n trials. Thus, this application for SAT procedure requires predicting information gain during the next block with multiple trials for each stimulus level and select the optimal —most informative stimulus level. We successfully implemented this "multiple-step ahead search"^16^ by computing the likelihood of k correct out of n trials.

Our simulation was carried out with the assumption that each SOA block consisted of 16 trials. The performance of the proposed Bayesian adaptive estimation (i.e., Procedure 4) would vary with the number of trials in each block, *block lengths*. Accuracy and precision at a given number of total trials can be improved by decreasing block length, especially when the number of total trials is limited. This is because the procedure with a shorter block length can acquire more information about the underlying SAT function by faster and more frequent switching to the stimulus level with the maximum information. To test the effect of the block length on the performance of the procedure, we ran additional simulations with four different settings of the block length (i.e., 1, 4, 16, and 64 trials in each block) and results supported the prediction. However, in practice, it should also be considered how frequently human observers can switch their decision criteria (from fast but inaccurate responses to slow but accurate responses), and how performance gets stabilized in an SAT experiment. It is not ascertained if participants successfully changed the psychological states every 16 trials. Thus, more studies are required to identify the optimal block length for the SAT procedure.

In our study, the functional form of SAT was the exponential approach to the limit (Eq. 1). The entire SAT function, often obtained with the response-signal procedure, provides rich information about the decision process at different decision criteria (or decision thresholds in context of the drift–diffusion model). In contrast, other process models for SAT (e.g., sequential sampling models including the drift–diffusion) mainly aim to investigate decision process on a single point on this function (i.e., a single decision criterion)^2,17^. For example, the parameter estimation in the drift model (e.g., decision time) is roughly positioned at a single point in the current exponential curve (each point at x-axis is related to a decision time). In the current SAT model, the parameters that define exponential function of diverse decision criteria were estimated.

The proposed procedure allows us to measure the SAT function with a relatively short experimental time without sacrificing accuracy and precision. In the simulation, we set the prior as a uniform distribution. The efficiency of the Bayesian adaptive procedure can be further improved by setting more realistic priors. Another improvement can be made for the assumption of expected RT for stimulus selection. We assumed that the expected response time, X, for a given SOA block is the time window's midpoint for simplicity. Indeed, expected response time could be longer than mid-point in short SOA blocks and shorter than mid-point in long SOA blocks. Elaboration on the expected RT can improve the efficiency of the optimal stimulus selection.

## Methods

### SAT model parametrization

In the SAT function of^2^, the probability of correct response p_i_, i.e., accuracy, has been modeled with a psychometric function of a given time constraint T_i_ and model parameters $\theta =\{\lambda ,\gamma ,\delta \}$ with the following equation.

3 $$ p_{i} = \Psi \left( {T_{i} ,\lambda ,\gamma ,\delta } \right) = \Psi_{\theta } \left( {T_{i} } \right) = \lambda \left( {1 - {\text{exp}}^{{ - \gamma \left( {T_{i} - \delta } \right)}} } \right) $$

The goal of the procedure is to estimate the parameters $\theta$ of a psychometric function $\Psi_{\theta } \left( {T_{i} } \right)$. The time constraint T_i_ could be controlled using the SOA of the response-signal. The prior probability distribution, θ, is defined as a three-dimensional joint probability distribution. Each dimension corresponds to each parameter of the SAT function.

The conventional approach fits the SAT curve using continuous accuracy data at a small number of SOA bins, as shown in Fig. 1a. In the current study, we propose a trial-based estimation of the SAT curve shown in Fig. 1b. The model parameter in the proposed method is estimated by the binarized response on the (semi-) continuous SOAs.

### Trial-based parameter estimation

**Figure 7:**
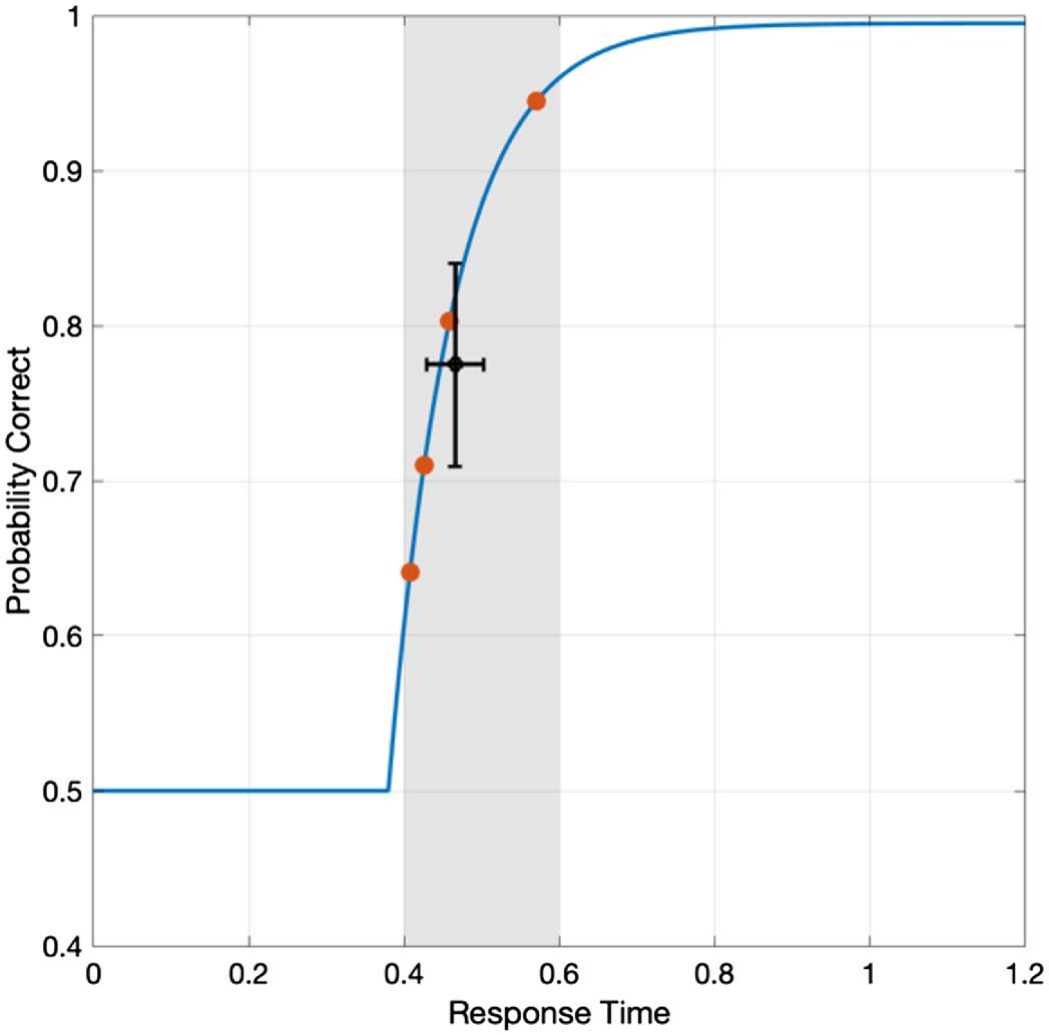
Illustration of a large variability problem. In a block of SOA = 0.4 s, RTs (red dots) are a random variable between 0.4 and 0.6 s (shaded area), and the mean RT has variability (horizontal error bar). The corresponding accuracy for the RTs also has large variability (vertical error bar) and measurement errors.

A unique feature of data analysis for an SAT experiment with response-signal manipulation is RT variability for each SOA condition. Because the mean RT is determined by the participant’s responses, the error for accuracy includes variability of RT and measurement error. (See Fig. 7 for the illustration of the large variability problem). Such variability results in an inaccurate and imprecise estimate of SAT function and its parameters. Therefore, estimation could be more efficient and robust if RT variability in each data point is reduced.

The procedure estimates $\theta =\left\{\lambda ,\gamma ,\delta \right\}$ of the SAT function using Bayesian inference^6,7,9–11^. It starts with a prior probability distribution ${p}_{0}(\theta ),$ and updates their posterior probability distribution based on the observer’s response after each trial.

After the t-th trial, the prior distribution $p_{t} \left( \theta \right)$ at the t-th trial is updated to the posterior distribution $p_{t} (\theta |r_{x} )$ with the observer’s binarized response $r_{{\text{x}}}$ (correct or incorrect) for a stimulus with a RT *x* by Bayes rule:

4 $$ p_{t} (\theta |r_{x} ) = \frac{{p_{t} \left( \theta \right)p(r_{x} |\theta )}}{{p_{t} \left( {r_{x} } \right)}} $$

where $p_{t} \left( \theta \right)$ and $p(r_{x} |\theta )$ are the prior probability density function and likelihood. The probability of a response $r_{{\text{x}}}$—either 0 or incorrect or 1 for correct—$p_{t} \left( {r_{x} } \right)$ for a stimulus with RT *x*, is estimated by weighting the empirical response probability by the prior:

5 $$ p_{t} \left( {r_{x} } \right) = \smallint p_{t} \left( \theta \right)p(r_{x} |\theta )d\theta $$

The likelihood of observing the response $r_{{\text{x}}}$ given θ and RT *x*, $p(r_{x} |\theta )$, can. be computed with the SAT function, $\Psi_{\theta } \left( x \right)$. The posterior at time *t* becomes the prior distribution of the subsequent trial at time *t* + *1*.

6 $$ p_{t + 1} = p_{t} (\theta |r_{{x_{t + 1} }}^{t + 1} ) $$

The parameter estimate after t-th trial is a marginal mean of the posterior distribution. Marginal posterior distributions of the parameters are computed via two-dimensional summation, and the expectations of the marginal posterior distributions are used to estimate the parameters of the SAT function after each trial. The estimated SAT function can be reconstructed by plugging the estimated parameters into the model (Eq. 2). The function also can be estimated by averaging reconstructed functions with parameters resampled from the posterior distribution.

We showed that the trial-based estimation of the SAT curve works successfully (see Results). Based on the trial-based Bayesian procedure, we then propose an adaptive optimal SOA selection scheme.

### Bayesian adaptive estimation with optimal stimulus selection

At the beginning of each SOA block, the procedure selects the most informative stimulus level (e.g., SOA) to capture the SAT function correctly. Most modern procedures for Bayesian parameter estimation select stimulus levels for the subsequent trial with the one-step-ahead search algorithm^6^. This algorithm computes the entropy of the posterior density for all stimulus levels, x, and responses for the subsequent trial, ${r}_{\mathrm{x}}$. Then, the expected posterior entropy is computed by weighting the likelihood of getting a response to the entropy of the posterior density. A new trial is chosen at an intensity that minimizes the expected posterior entropy with respect to X^6,7,9,10^. Alternatively, the stimulus selection can be made by maximizing the expected information gain, quantified as the entropy change between the prior and posterior (see Heitz^17^).

However, in SAT experiments, stimulus level changes after each block, rather than after each trial. Since RT has variability in each SOA block of SAT experiment with the response-cue approach, we assume that the expected response time, X, for a given SOA block is the midpoint of a time window for simplicity. For example, the expected response time at t-th block, X_i_ = 0.6 + 0.2/2 = 0.7 for 0.6 s of SOA block when the response window is 0 to 0.2 s.

The expected information gain after the next block can be computed by mutual information:

7 $$ I_{t} \left( {R_{x} ;\Theta } \right) = H_{t} \left( {R_{x} } \right) - H_{t} (R_{x} |\Theta ) $$

For the case in which n trials are included in a block, entropy can be defined for all types of responses, as a function of x,

8 $$ H_{t} (R_{x} |\theta ) = - \mathop \sum \limits_{{r \in \left\{ {0,1, \cdots ,n} \right\}}} p_{t} (r_{x} |\theta ){\text{log}}p_{t} (r_{x} |\theta ) $$

where

9 $$ p(r_{x} |\theta ) = p(r = k|x,\theta ) = \left( {\begin{array}{*{20}c} n \\ k \\ \end{array} } \right)\Psi_{\theta } (x)^{k} (1 - \Psi_{\theta } \left( x \right))^{n - k} $$

10 $$ H_{t} (R_{x} |\Theta ) = \mathop \sum \limits_{\theta } p_{t} \left( \theta \right)H(R_{x} |\theta ) $$

11 $$ H_{t} (R_{x} |\theta ) = - \mathop \sum \limits_{{r \in \left\{ {0,1, \cdots ,n} \right\}}} p_{t} (r_{x} |\theta ){\text{log}}p_{t} (r_{x} |\theta ) $$

The SOA for the next block is chosen at an intensity $x_{t + 1}$ that maximizes the expected information gain $I_{t} \left( {\Theta ;R_{x} } \right)$ for x.

The procedure stops after when precision reached a target level or after a predefined number of trials. Our study showed that 50–200 trials were sufficient to achieve an appropriate level of precision. The MATLAB code is available for download at a public repository (https://osf.io/75sqe).

### Simulation and comparison of model estimation procedures

The performance of the procedure for observers with a range of parameters of SAT function was assessed by simulating four virtual observers with different values. To evaluate the benefit of the Bayesian adaptive SAT procedure (Procedure 4) and trial-by-trial fitting procedure (Procedures 2 and 3) over the conventional SAT procedure with the MCS (Procedure 1), we compared the accuracy and precision of suggested procedures.

#### Simulated experimental setting

##### Procedure 1–3

The parameters of the simulated observers are summarized in Table 1. The parameters of simulated observers were set based on our pilot studies of the Stroop task and Flanker task. The simulated observers performed a 2-alternative forced-choice (2AFC) task employing the response-signal manipulation. Observers’ responses for RT and correctness were simulated for 8 SOA levels (0.06, 0.09, 0.12, 0.24, 0.36, 0.48, 0.60, and 1.20 s) with the time window for response of 0.2 s. In each trial, response time, x, for all observers were generated randomly from an ex-Gaussian distribution^18^ (μ = 0.3, σ = 0.06, τ = 0.08), which was constrained by SOA of the block and the time window. The expected probability of correct, pc(x), of the simulated observer was calculated for the simulated RT, x. Observer’s response in each trial was simulated by drawing a random number r from a uniform distribution over the interval from 0 to 1. The response was labeled as correct if r < pc(x) and incorrect otherwise. Each simulated experimental run consisted of 2048 trials (= 256 trials per SOA condition X 8 SOAs). Simulated runs were iterated 10,000 times for each observer.

**Table 1:** SAT parameters of simulated observers.

	λ	γ	δ
Observer 1	0.470	7.5	0.28
Observer 2	0.450	5.0	0.22
Observer 3	0.495	22	0.24
Observer 4	0.495	20	0.36

##### Procedure 4

We simulated the same four observers’ responses as in Procedures 1–3. Stimulus space (i.e., possible cue delays) was sampled from 0 to 1.2 s with 49 equally spaced samples. Each block's stimulus was selected by the observer’s responses and the maximum information gain rule (described in the previous Method section). Each simulated experimental run consisted of 2048 trials (= 16 trials per block X 128 blocks). Simulated runs were iterated 10,000 times for each observer.

#### Model parameter estimation methods

##### Procedure 1: The conventional SAT function estimation with averaging RT and correctness

The traditional SAT experiment uses constant stimuli (MCS): stimuli are presented for a fixed set of predetermined SOAs repeatedly in random order. After the experiment is completed, RT and correctness were averaged for each SOA condition for the data analysis. Alternatively, all responses can be grouped by RT into serval bins, then the mean correctness, accuracy is calculated for each bin. The mean RT and accuracy for SOA or RT bins were fitted to the model using the maximum likelihood estimation (MLE) or the least square estimation (LSE).

##### Procedure 2: The maximum likelihood SAT function estimation with trial-by-trial RT and correctness

A variation of the SAT estimation procedure could be fitting trial-by-trial RT and correctness to the model without grouping and averaging by RT or SOA. In this procedure, the model was fitted with trial-by-trial RT and correctness using the proposed binarized scheme, without averaging RT and correctness by SOA or RT. All data collection procedures were the same as Procedure 1: experiment with MCS and fitting with MLE/LSE after the data collection.

##### Procedure 3: The Bayesian SAT function estimation with trial-by-trial RT and correctness

The model was fitted to the same simulated dataset with trial-by-trial RT and correctness in Procedure 2 but using the Bayesian estimation procedure. The prior was set to a uniform distribution over 21 linearly spaced λ values (from 0.4 to 0.5), 30 linearly spaced γ values (from 1 to 30), and 25 linearly spaced δ values (from 0.02 to 0.5).

##### Procedure 4: Bayesian estimation with trial-by-trial RT and correctness

In this procedure, we used an adaptive approach for estimating SAT function with Bayesian inference and optimal stimulus selection based on procedure 3. The SAT function is characterized by an exponential model and a prior distribution of the parameters. The stimulus level (i.e., SOA) for the next block is selected to maximize the expected information gain on the SAT function parameters. After each trial in each block, the parameters' posterior distribution is updated using the Bayes rule and observer’s response. Stimulus selection and posterior update are repeated until a fixed number of blocks or a pre-set test precision is reached. In this section, we described the algorithm of the procedure and evaluated the performance of the procedure with a batch of simulations.

The parameter space and the prior were set to the same as in Procedure 3. The SAT function was estimated by the resampling method: the procedure resamples the posterior distribution of the parameters 1000 times, reconstructs the SAT functions from each set of sample parameters, and then averages over all the resampled SAT functions.

All four procedures are summarized in Table 2. The details will be discussed in the following sections.

**Table 2:** Procedures for estimating SAT functions and their parameters.

	Data collection		Data analysis		
	Stimulus selection	Stimulus change	Data	Model fitting	Estimation frequency
Procedure 1	MCS	By long block	Averaged	MLE/LSE	Offline
Procedure 2	MCS	By short block	Trial-by-trial	MLE/LSE	Offline
Procedure 3	MCS	By short block	Trial-by-trial	Bayesian	Online
Procedure 4	Adaptive	By short block	Trial-by-trial	Bayesian	Online

MCS: method of constant stimuli, MLE: maximum likelihood estimation, LSE: least square estimation.

#### Performance evaluation

We compared the fitting performance—accuracy and precision—of fitting procedures. A good procedure should quickly increase the accuracy and precision of the estimated SAT function or its parameters as the trial number increases. We assessed bias of estimated SAT parameters and function by mean absolute difference between true and estimated parameters or accuracy and precision by the standard deviation of repeated measures^19,20^.

Accuracy can be defined by the inverse of bias. For each parameter, the bias of estimate was calculated by the mean absolute discrepancy between the estimated and true parameter:

12 $$ bias_{i} = \frac{{\mathop \sum \nolimits_{j = 1}^{J} \left| {P_{ij} - P_{true} } \right|}}{J} $$

where P_true_ is the true parameter value, and P_ij_ is the parameter estimate obtained after the i-th trial in the j-th simulation.

Precision is assessed by the inverse of the standard deviation of repeated measures. It is also important to evaluate and compare the convergence of estimated SAT functions to the true function and its parameters of two procedures. The average absolute bias of the estimated SAT function can be calculated as:

13 $$  average\;absolute\;bias_{i}  = \frac{{\sum\nolimits_{{k = 1}}^{K} {\left| {\sum\nolimits_{{j = 1}}^{J} {(Pc_{{ij}}^{k}  - Pc_{{true}}^{k} )} } \right|} }}{{J \times K}},  $$

where $Pc_{ij}^{k}$ is the estimated probability correct for expected RT after i-th trials obtained in the mean RT in k-th SOA block of the j-th simulation and $Pc_{true}^{k}$ is the true probability correct. Since mean RT’s vary across simulations, we computed expected pc for the fixed set of time points (0.06, 0.09, 0.12, 0.24, 0.36, 0.48, 0.60, and 1.20 s) with estimated SAT function for all simulations. The precision of the estimated SAT function was assessed by the averaged standard deviation of repeated measures over SOAs:

14 $$ SD_{i} = \sqrt {\frac{{\mathop \sum \nolimits_{k = 1}^{K} \mathop \sum \nolimits_{j = 1}^{J} \left( {Pc_{ij}^{k} - mean(Pc_{ij}^{k} } \right))^{2} }}{J \times K}} $$

In Bayesian estimation, precision also can be assessed by the half-width of the credible interval (HWCI) of the posterior distribution^21^. The HWCI refers to the shortest interval that covers most of the distribution. The 95% credible interval represents a 95% probability that the actual value lies within the range^22^, whereas the confidence interval, the most popular index of precision, represents an interval that contains the true value of the parameter for 95% of unlimited repetitions^23^.

### Ethical approval

Not applicable to the current study. The current study is based on simulation.

## Data Availability

No datasets were generated or analysed during the current study.

## References

[1] Reed AV (1973). Speed-accuracy trade-off in recognition memory. Science.

[2] Wickelgren WA (1977). Speed-accuracy tradeoff and information processing dynamics. Acta Psychol..

[3] Baek J, Lesmes LA, Lu ZL (2016). qPR: An adaptive partial-report procedure based on Bayesian inference. J. Vis..

[4] Kim W, Pitt MA, Lu ZL, Steyvers M, Myung JI (2014). A hierarchical adaptive approach to optimal experimental design. Neural Comput..

[5] King-Smith PE, Grigsby SS, Vingrys AJ, Benes SC, Supowit A (1994). Efficient and unbiased modifications of the QUEST threshold method: Theory, simulations, experimental evaluation and practical implementation. Vision Res..

[6] Kontsevich LL, Tyler CW (1999). Bayesian adaptive estimation of psychometric slope and threshold. Vis. Res..

[7] Kujala JV, Lukka TJ (2006). Bayesian adaptive estimation: The next dimension. J. Math. Psychol..

[8] Leek MR (2001). Adaptive procedures in psychophysical research. Percept Psychophys..

[9] Cobo-Lewis AB (1997). An adaptive psychophysical method for subject classification. Percept. Psychophys..

[10] Lesmes LA, Lu Z-L, Baek J, Albright TD (2010). Bayesian adaptive estimation of the contrast sensitivity function: the quick CSF method. J. Vis..

[11] Watson AB, Pelli DG (1983). QUEST: A Bayesian adaptive psychometric method. Percept Psychophys..

[12] Dorr M (2015). Next-generation vision testing: The quick CSF. Current Direct. Biomed. Eng..

[13] Hou F (2010). qCSF in clinical application: efficient characterization and classification of contrast sensitivity functions in amblyopia. Invest. Ophthalmol. Vis. Sci..

[14] Hou F, Lesmes LA, Bex P, Dorr M, Lu Z-L (2015). Using 10AFC to further improve the efficiency of the quick CSF method. J. Vis..

[15] Lesmes LA (2015). Developing Bayesian adaptive methods for estimating sensitivity thresholds (d') in Yes-No and forced-choice tasks. Front. Psychol..

[16] Gu, H., Myung, J. I., Pitt, M. a. & Lu, Z.-l. (eds M. Knauff, M. Pauen, N. Sebanz, & I. Wachsmuth) 2452–2457 (Cognitive Science Society, 2013).

[17] Heitz RP (2014). The speed-accuracy tradeoff: History, physiology, methodology, and behavior. Front Neurosci.

[18] Van Zandt T (2000). How to fit a response time distribution. Psychon. Bull. Rev..

[19] Lu, Z.-L. & Dosher, B. A. *Visual Psychophysics: From Laboratory to Theory*. (The MIT Press, 2013).

[20] Treutwein B (1995). Adaptive psychophysical procedures. Vis. Res..

[21] Edwards W, Lindman H, Savage LJ (1963). Bayesian statistical inference for psychological research. Psychol. Rev..

[22] Clayton, D. & Hills, M. *Statistical models in epidemiology*. (Oxford University Press, 2013).

[23] Rothman, K. J. & Greenland, S. *Modern Epidemiology*. 2nd edn, (Lippincott Williams & Wilkins, 1998).

